# The Role of PGC-1α/UCP2 Signaling in the Beneficial Effects of Physical Exercise on the Brain

**DOI:** 10.3389/fnins.2019.00292

**Published:** 2019-03-29

**Authors:** Viviane José de Oliveira Bristot, Ana Cristina de Bem Alves, Liziane Rosa Cardoso, Débora da Luz Scheffer, Aderbal Silva Aguiar

**Affiliations:** ^1^Research Group on Biology of Exercise, Department of Health Sciences, Centro Araranguá, Federal University of Santa Catarina, Araranguá, Brazil; ^2^Laboratório de Bioenergética e Estresse Oxidativo, Departamento de Bioquímica, Centro de Ciências Biológicas, Universidade Federal de Santa Catarina, Florianópolis, Brazil

**Keywords:** physical exercise, mitochondria, irisin, FNDC5, PGC-1α, UCP2

## Abstract

In understanding the pathology of neurological diseases, the role played by brain energy metabolism is gaining prominence. Animal models have demonstrated that regular physical exercise improves brain energy metabolism while also providing antidepressant, anxiolytic, antioxidant and neuroprotective functions. This review summarizes the latest evidence on the roles played by peroxisome proliferator-activated receptor gamma (PPAR-γ) coactivator 1-alpha (PGC-1α) and mitochondrial uncoupling protein (UCP) in this scenario. The beneficial effects of exercise seem to depend on crosstalk between muscles and nervous tissue through the increased release of muscle irisin during exercise.

## Introduction

A physically inactive lifestyle is associated with the development of non-communicable diseases (NCD), such as cardiovascular diseases, type 2 diabetes, some cancers, and an overall increased mortality rate (Booth et al., 2012; Koster et al., 2012; Biswas et al., 2015; Same et al., 2016; Patterson et al., 2018). Physical inactivity is also considered a risk factor for abdominal obesity, high serum triglyceride levels, low-density lipoprotein, cholesterol, hypertension, and hyperglycemia, which together characterize metabolic syndrome (Bankoski et al., 2011). Physical exercise has several benefits for physical and mental health (Aguiar et al., 2008; Garber et al., 2011; Esteban-Cornejo et al., 2015; Loprinzi, 2015), including increased physical and cardiorespiratory capacity (or fitness), improved body composition and balance (or fatness), and greater muscle strength and flexibility (Garber et al., 2011; Geneen et al., 2017). Physical exercise also improves the serum lipid profile, decreases glucose intolerance, and attenuates insulin resistance (Lin et al., 2015; Brymer and Davids, 2016; Qiu et al., 2018). The literature supports the fitness-fatness hypothesis, which suggests that a higher level of cardiorespiratory fitness will reduce the adverse effects of obesity on morbidity and mortality, making obesity a much less important factor for health than is generally believed (Hainer et al., 2009; Fogelholm, 2010; Barry et al., 2014). The data are mixed, but for many authors, fitness is more important than fatness for early mortality (Blair et al., 1989; Wei et al., 1999; McAuley et al., 2009; Barry et al., 2014). This is important for individuals who are unable to lose weight but are able to engage in a regular physical activity program.

The American College of Sports Medicine (ACSM) recommends increasing total energy expenditure (TEE; kcal/day) for health, with a minimum of 30 min of moderate physical exercise 5 days/week or 20 min of vigorous exercise 3 days/week (Haskell et al., 2007). The ACSM also recommends combining moderate (3–6 MET) and vigorous activities (>6 MET) (Haskell et al., 2007). The World Health Organization (2000) recommends that individuals participate in at least 150 or 75 min/week of moderate or vigorous physical activities, respectively. For overweight and obesity, the ACSM recommends increased physical activity, between 150 and 250 min/week to prevent weight gain or provide modest weight loss (Donnelly et al., 2009). Larger amounts of exercise (>250 min/week) are needed for clinically significant weight loss (Donnelly et al., 2009). However, physical activity for weight loss is controversial; the amount of weight lost due to an exercise intervention is often less than what is predicted to be lost based on the exercise workload, suggesting a smaller increase in TEE (and smaller energy imbalance) than expected (Thomas et al., 2012; Melanson et al., 2013; Flack et al., 2018). This reduced energy imbalance occurs through metabolic and behavioral modifications in humans (Pontzer, 2018) and reinforces the hypothesis that fitness is more important than fatness for health. In general, the 150 min/week of moderate physical activity or 60–75 min/week of vigorous activity recommendations of ACSM and WHO is effective for overall health.

Even single exercise sessions, which increases the production of endogenous opioids (Geneen et al., 2017), angiogenesis factors (such as vascular endothelial growth factor [VEGF], hypoxia-induced 1 alpha factor [HIF-1α] and erythropoietin [EPO]) (Ribeiro et al., 2017) appear to be healthy, and they protect against hyperglycemia peaks (Lang Lehrskov et al., 2018) in humans. A single exercise session increases the plasma endocannabinoid levels in mice (Fuss et al., 2015), which is a possible mechanism for the euphoric state (runner’s high) that occurs after long runs (Boecker et al., 2008).

The central nervous system (CNS) was the last physiological system approached by the exercise sciences. Lack of exercise is a major cause of chronic diseases (Booth et al., 2012), including brain diseases, such as depression (Farmer et al., 1988; Aguiar et al., 2014), and neurodegenerative diseases (Radak et al., 2010; Xu et al., 2010; Aguiar et al., 2016). However, physical exercise is a neuroprotective agent against depression (Schuch et al., 2017), anxiety disorders (Jayakody et al., 2014), cognitive decline/dementia in elderly people (Aguiar et al., 2011; Shen et al., 2016), Parkinson’s disease (Chen et al., 2018), and Alzheimer’s disease (Aguiar et al., 2016; Law et al., 2018). Animal studies have shown that physical exercise increases neuronal survival, cerebral vascularization, neurogenesis, and mitochondrial metabolism, while it decreases the effects of neurotoxins on the CNS (Aguiar et al., 2014; Zhang and Zhang, 2016). Iris and uncoupling proteins (U) are candidate mechanisms for these exercise-induced changes.

**FIGURE 1:**
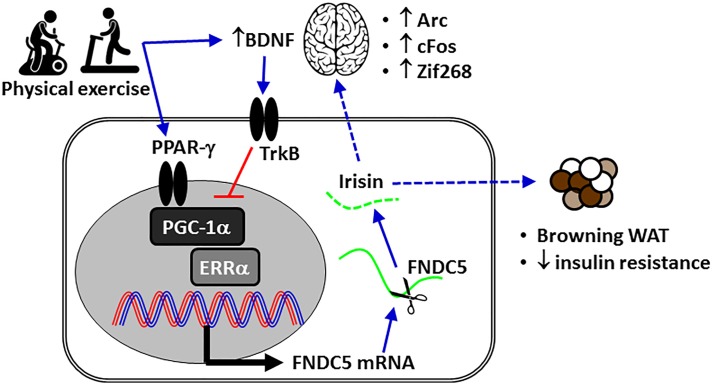
The mechanism of action of irisin in metabolism-associated health issues or metabolic diseases. The expression of Arc, cFos, and Zif268 is induced by neuronal activity. BDNF, brain-derived neurotrophic factor; ERRα, estrogen-related receptor alpha; FNDC5, fibronectin domain-containing protein 5; PGC-1α, peroxisome proliferator-activated receptor gamma coactivator-1-alpha; TrkB, tyrosine receptor kinase B; WAT, white adipose tissue.

**FIGURE 2:**
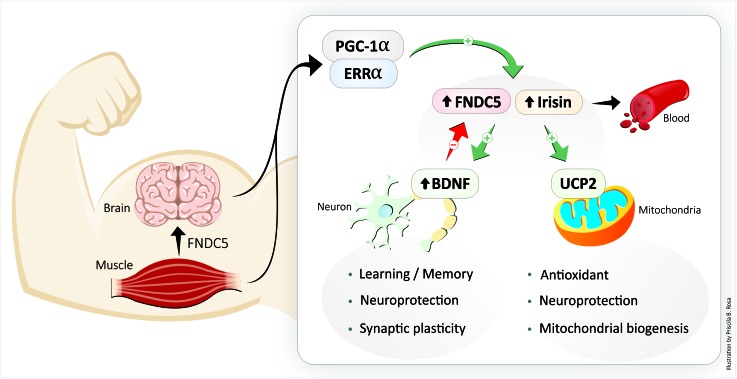
The exercise-increased circulating protein irisin links structural and functional modifications in muscle and brain. BDNF, brain-derived neurotrophic factor; FNDC5, fibronectin type III domain-containing protein 5; UCP2, uncoupling protein 2.

In mammals, transcriptional peroxisome proliferator-activated receptor gamma (PPAR-γ) coactivator 1-alpha (PGC-1α)/fibronectin type III domain-containing protein 5 (FNDC5, the precursor of irisin), which is secreted during exercise, promotes the browning of beige fat cells in white adipose tissue (Figure 1), resulting in enhanced thermogenesis and increased energy expenditure (Hofmann et al., 2014). In the CNS (Figure 2), FCDN5/irisin regulates central mechanisms that mediate adaptive responses by (a) improving neuronal mitochondrial decoupling and (b) increasing the expression of neurotrophins and neuroprotective proteins such as neuronal PAS domain protein 4 (NPAS4), cFOS, activity-regulated cytoskeleton-associated protein (ARC), and zinc finger protein 268 (ZIF268) (Figure 1; Wrann et al., 2013; Wrann, 2015). In brown adipose tissue, mitochondrial uncoupling is effected by a specific protein, referred to as uncoupling protein-1 (UCP1), in the inner mitochondrial membrane (Ricquier and Bouillaud, 2000). The cloning of UCP2 and UCP3, two homologs of UCP1, has boosted research into the importance of respiration control in metabolic processes, metabolic diseases and energy balance (Ricquier and Bouillaud, 2000). PPAR-γ/PGC-1α expression also improves mitochondrial decoupling, which reduces mitochondrial membrane potential and reactive oxygen species (ROS) production, oxidative damage, mitochondrial calcium overload and potential apoptotic events through the induction of uncoupling protein 2 (UCP2) (Andrews et al., 2005). Therefore, FND5/irisin is essential for processes involving neurotrophins and synaptic plasticity, mitochondrial biogenesis, and resistance to neuronal stress (Wrann et al., 2013; Marosi and Mattson, 2014; Raefsky and Mattson, 2017).

The purpose of this manuscript is to review the roles played by FND5/irisin and UCP2, which are important for energy metabolism, in the neuroprotective and antioxidant effects of physical activity in the CNS.

## The Exercise-Induced Release of Irisin and Its Neuroprotective Effects

Because irisin is an exercise-induced hormone (or myokine), it is unclear whether the physical exercise-related CNS benefits are attributable to irisin. Irisin is a 112 amino acid peptide that is cleaved (by an unknown protease) from the glycosylated type I membrane protein FNDC5 and released into the bloodstream in a PGC-1α-dependent manner through a muscle contraction-mediated transcription mechanism (Bostrom et al., 2012). PGC-1α is a transcriptional coactivator and does not bind to DNA directly; it needs to interact with another transcription factor to induce neuronal FNDC5 gene expression (Xu, 2013). Several clues indicate that the PGC-1α binding partner is orphan nuclear estrogen-related receptor alpha (ERRα) (Kamei et al., 2003; Xu, 2013). Moreover, irisin enhances PGC-1α expression in the hippocampus and prefrontal cortex of mice in a positive feedback loop (Siteneski et al., 2018). The irisin released from muscles is a myokine that acts preferentially on the subcutaneous ‘beige’ fat and causes it to ‘brown’ by increasing the expression of UCP1 and other thermogenic genes (Bostrom et al., 2012). Irisin is involved in human biological adaptations such as increased muscle strength, decreased obesity and insulin resistance, and also has physical and psychological benefits (Bostrom et al., 2012; Ghahrizjani et al., 2015). Currently, it is hypothesized that irisin circulates in the blood in vesicles containing other molecules, such as proteins, miRNA and nucleic acids, until reaching the target tissues, which include adipose tissue and the brain (Safdar et al., 2016). The exercise-induced release of peptides and nucleic acids from skeletal muscle (collectively termed ‘exerkines’) has been implicated in mediating systemic biological adaptations (Safdar et al., 2016).

The contraction of large muscle groups increases the muscle-specific expression of PGC1-α and FNDC5 and consequent release of irisin (Bostrom et al., 2012). In humans, blood irisin levels reach approximately 3.6 ng/ml in sedentary individuals and 4.3 ng/ml in active individuals after 12 weeks of regular aerobic exercise (Jedrychowski et al., 2015). Irisin contributes to exercise-induced physiological adaptations in the cardiovascular, immune, digestive, and adipose systems (Bostrom et al., 2012; Zhang et al., 2015; Mazur-Bialy et al., 2017). In obese adult humans, exercise combined with caloric restriction improves health (Mozaffarian et al., 2011) and increases concentrations of circulating irisin (Huang et al., 2017). Although skeletal muscle is the major source of exercise-induced irisin that is released into the plasma (Roca-Rivada et al., 2013), it remains unclear whether neuronal irisin is derived from muscles or is produced in neurons. In neurons, PGC-1α interacts with estrogen-related receptor alpha (ERRα) to regulate the expression of FNDC5 (Figure 1; Wrann et al., 2013). Moreover, the increased expression of FNDC5 promotes neuronal development and differentiation (Forouzanfar et al., 2015; Ghahrizjani et al., 2015). FNDC5 can be found in the cerebrospinal fluid, cortical neurons, paraventricular neurons in the hippocampus, Purkinje cells in the cerebellum, hypothalamus, multipolar neurons in the anterior nerve of the spinal cord, and in astrocytes and microglia in the cerebral tissue (Dun et al., 2013; Moon et al., 2013; Piya et al., 2014; Albayrak et al., 2015). In the rat H19-7HN cell line, irisin (50–100 nmol/l) increased the proliferation of hippocampal cells, thus reinforcing its role in neurogenesis (Moon et al., 2013).

Wrann et al. (2013) demonstrated that the expression of PGC-1α and FNDC5 in the hippocampal neurons was enhanced after the mice spent 2 weeks running in exercise wheels. Higher FNDC5 expression also increased the expression of the genes BDNF, Arc, cFos, and Zif268, which is induced by neuronal activity. FNDC5 expression is counterbalanced by BDNF expression in a negative feedback mechanism (Figure 1; Wrann et al., 2013). It is possible that this feedback loop is a CNS detraining mechanism that requires regular exercise to maintain its neurological benefits. This evidence suggests that the induction of FNDC5 is part of the transcriptional response to exercise, including neuroplasticity and neuroprotection, in the CNS. Exercise-induced PGC-1α and irisin reduced ischemia-induced neuronal injury (Zhang et al., 2012) via activation of the Akt and ERK1/2 signaling pathways in mice (Li et al., 2017). Exercise-induced irisin also reduced the brain infarct volume, neurological deficits, brain edema and the body weight decline of mice subjected to middle cerebral artery occlusion (MCAO) (Li et al., 2017). Since BDNF is a critical regulator of neural plasticity, irisin may act as a key regulator of neuronal survival following cerebral ischemia. Physical activity (running wheel, 12 weeks) increases levels of circulating irisin and BDNF even in 20-month-old female rats (Belviranli and Okudan, 2018), increases the expression of BDNF and decreases neuroinflammation in the hippocampus of aged rats and mice, and has motor and cognitive benefits (Aguiar et al., 2011; Dallagnol et al., 2017).

Exercise is an antidepressant (Blumenthal et al., 1999; Cunha et al., 2013), and irisin has been linked to the antidepressant effects of exercise. Reduced irisin levels are associated with mood impairment and reduced BDNF levels in humans (Papp et al., 2017; Szilasi et al., 2017), and increased circulating concentrations of irisin have been shown to have antidepressant effects in mice (Siteneski et al., 2018). A possible mechanism for the antidepressant effect is the activation of the PGC-1α/BDNF pathway by irisin after exercise (Wrann et al., 2013). As previously mentioned, BDNF is a critical neurotrophin involved in the differentiation, survival, maintenance, and function of neurons; it is also involved in learning and memory processes (Wrann et al., 2013). Torma et al. (2014) demonstrated that the neurotrophic role of BDNF is dependent on PGC-1α.

In humans, the increased plasma levels of FNDC-5, irisin and BDNF seem to depend on exercising large muscle groups, as can be achieved with regular Nordic walking training (Gmiat et al., 2018), aquarobics (16 weeks) (Kim and Kim, 2018), and CrossFit training (12 weeks) (Murawska-Cialowicz et al., 2015). The electrical stimulation of small muscle groups increases BDNF but not irisin in the hippocampus of rats (Maekawa et al., 2018). In healthy elderly women, Nordic walking training improved body composition, anaerobic capacity and cardiovascular fitness (Gmiat et al., 2018), and CrossFit training improved psychological (Quality-of-Life Assessment and The Beck Depression Inventory-2) and cognitive functions (D2 test of attention and Trail Making Test A&B).

## Neuronal UCP2 –An Antioxidant Mechanism of Exercise

The expression of neuronal uncoupling proteins (UCP) is induced by metabolic and oxidative challenges such as physical exercise and caloric restriction (Liu et al., 2015). UCP facilitates proton flux through the internal mitochondrial membrane, thereby dissociating the oxidative phosphorylation of ATP synthesis (Wei et al., 2009). The enhanced proton flux process reduces the mitochondrial membrane potential, increases mitochondrial respiration, decreases the ATP/ADP ratio, and dissipates chemical energy in the form of heat (Chu et al., 2009). Acute mitochondrial decoupling reduces mitochondrial ATP production; however, chronic mitochondrial decoupling promotes an increase in the number of mitochondria and an increased level of ATP production (Coppola et al., 2007).

Initially, UCP1, which functions in heat production, was identified in brown adipose tissue (Geisler et al., 2017). UCP2 is found in organs and tissues such as the liver, kidney, pancreas, endothelium, immune cells, and the CNS (Pecqueur et al., 2001; Chu et al., 2009). UCP2, UCP4, and UCP5 are expressed in the CNS, are referred to as neuronal U, and are involved in the adaptation to cellular stress (Chu et al., 2009). The distribution of neuronal UCPs demonstrates the relevance of mitochondrial decoupling in the CNS to the control of neuronal, neuroendocrine, and autonomic responses (Richard et al., 1998). UCP2 is expressed in the hypothalamus, especially in the arcuate nucleus, limbic system, cerebellum, choroid plexus, and encephalic trunk (Richard et al., 1998; Arsenijevic et al., 2000). UCP4 is detected in most brain tissues, but it is expressed at lower levels in the spinal cord and *Substantia nigra* (Mao et al., 1999). UCP5 is expressed in the cerebral cortex, hippocampus, thalamus, hypothalamus, amygdala, basal ganglia, and spinal cord (Kwok et al., 2010).

Neuronal UCPs influence the regulation of mitochondrial biogenesis, calcium flux, ROS production, and local temperature (Teshima et al., 2003). Neuronal UCPs play an important role in the reduction of ROS production and consequent reduction in oxidative stress without compromising the production of ATP (Arsenijevic et al., 2000). Exposure of cultured neurons to decoupling agents, such as carbonyl cyanide-4-(trifluoromethoxy) phenylhydrazone (FCCP) or 2,4-dinitrophenol (2,4-DNP or simply DNP), reduces the mitochondrial membrane potential and inhibits mitochondrial calcium absorption, and thus prevents cell death (Stout et al., 1998). Neuronal UCPs also influence the temperature of neuronal microenvironments and thus contribute to the dynamics of neuronal activity through greater synaptic plasticity and neuronal transmission (Arsenijevic et al., 2000). Some studies have suggested that mitochondrial decoupling is linked to neuroprotection against physiological processes and pathological mechanisms including aging, Alzheimer’s and Parkinson’s diseases, neuronal hypoxia and ischemia, and epilepsy (Bechmann et al., 2002; Dietrich et al., 2008).

Among the neuronal UCPs, UCP2 is involved in central autonomic, endocrine, and metabolic regulation and is thus associated with cognition, mood, and behavior (Diano et al., 2000; Wang et al., 2014). UCP2 in the ventromedial nucleus restores glucose tolerance and regulates insulin sensitivity mediated by glucose-excited neurons, which is important for the physiological control of systemic glucose metabolism (Toda et al., 2016). In the arcuate nucleus, UCP2 is associated with mitochondrial fission, increased mitochondrial density and diminished mitochondrial size (Toda et al., 2016). UCP2 shows increased expression after neuronal injury (Bechmann et al., 2002). UCP2 induces mitochondrial decoupling in nigral neurons of the substantia nigra pars compacta (SNpc) and can prevent the loss of dopaminergic cells after 1-methyl-4-phenyl-1,2,5,6-tetrahydropyridine (MPTP)-induced toxicity, which is an essential effect to delay Parkinson’s disease pathophysiology (Echtay et al., 2001; Horvath et al., 2003). The relation of UCP2 to exercise occurs through the PGC-1α/PPARα pathway, which can regulate neuronal UCP2 (Wu et al., 2014) and BDNF (Gomez-Pinilla et al., 2008). Physical activity (running wheel, 4 weeks) increased UCP2 expression and mitochondrial oxygen consumption in coupled and uncoupled mitochondria in the hippocampus of mice (Dietrich et al., 2008). Moreover, physical activity (running wheel, 1 week) and exercise (treadmill, 12 weeks) increased UCP2 levels in the hippocampus, cerebellum and brain cortex mitochondria of adult rats (Gomez-Pinilla et al., 2008; Marques-Aleixo et al., 2015). The exercise-induced (running wheel, 1 week) increase in UCP2 correlated with increased BDNF in the hippocampus of rats (Gomez-Pinilla et al., 2008). These changes in BDNF content and mitochondrial metabolism (Vaynman et al., 2006; Aguiar et al., 2007, 2014) coincided with an increase in the number of mitochondria and dendritic spine synapses in the granule cells of the dentate gyrus and the stratum radiatum of the CA1 region and were dependent on UCP2 expression because such changes were not observed in UCP2 knockout mice (Dietrich et al., 2008). The absence of proper mitochondrial decoupling reduced the number of synapses in hippocampal neurons due to the increase in free radical production in response to exercise, thus demonstrating the characteristic protective effect of UCP2 in this knockout mouse model (Dietrich et al., 2008). For example, doxorubicin is an effective antineoplastic agent that is limited by mitochondrial toxicity in non-target tissues, including the brain (Marques-Aleixo et al., 2016; Flanigan et al., 2018). Doxorubicin (2 mg/kg, i.p.) impaired spatial learning/memory and decreased UCP2 protein content in cerebellum and brain cortex mitochondria of adult rats, both of which were prevented by physical activity (treadmill, 12 weeks) (Marques-Aleixo et al., 2016). The UCP2-related nuclear respiration factor 1 (NRF1) and mitochondrial transcription factor A (TFAM) genes, which are involved in mitochondrial biogenesis, are associated with synaptic plasticity and decreased neuronal vulnerability to cellular stress (Simon-Areces et al., 2012). In an animal model of Parkinson’s disease, 8 weeks of treadmill exercise stimulated mitochondrial biogenesis and increased NRF2 and TFAM expression in the striatum of mice, which protected against neuronal death caused by the neurotoxin 6-OHDA (Aguiar et al., 2016). The mitochondrial mechanism related to UCP2 function is essential for the appropriate bioenergetic adaptation of neurons to increased neuronal activity and synaptic plasticity in response to physical activity.

## Conclusion

Exercise improves the PGC-1α/BDNF pathway (muscle/brain) through the signaling of circulating irisin, which strengthens synapses and exhibits neuroprotective and antidepressant effects. These neuroprotective effects of exercise are enhanced by the antioxidant effects of UCP2, which is expressed at increased levels in neurons in response to exercise. Therefore, the evidence suggests a role for irisin/UCP2 in the mechanism underlying the benefits of physical exercise on the CNS. Consequently, irisin/UCP2 might be a potential therapeutic target to improve brain function and prevent or treat neurological and neurodegenerative diseases.

## Author Contributions

All authors listed have made a substantial, direct and intellectual contribution to the work, and approved it for publication.

## Conflict of Interest Statement

The authors declare that the research was conducted in the absence of any commercial or financial relationships that could be construed as a potential conflict of interest.
